# Antimicrobial Activity of the Quinoline Derivative HT61 against Staphylococcus aureus Biofilms

**DOI:** 10.1128/AAC.02073-19

**Published:** 2020-04-21

**Authors:** C. J. Frapwell, P. J. Skipp, R. P. Howlin, E. M. Angus, Y. Hu, A. R. M. Coates, R. N. Allan, J. S. Webb

**Affiliations:** a School of Biological Sciences, Faculty of Environmental and Life Sciences, University of Southampton, Southampton, United Kingdom; b National Biofilms Innovation Centre, University of Southampton, Southampton, United Kingdom; c Institute for Life Sciences, University of Southampton, Southampton, United Kingdom; d Centre for Proteomic Research, Institute for Life Sciences, University of Southampton, Southampton, United Kingdom; e Biomedical Imaging Unit, Southampton General Hospital, Southampton, United Kingdom; f Institute of Infection and Immunity, St George’s, University of London, London, United Kingdom; g Helperby Therapeutics Group plc, London, United Kingdom

**Keywords:** HT61, *Staphylococcus aureus*, antibiotics, biofilms, proteomics

## Abstract

Staphylococcus aureus biofilms are a significant problem in health care settings, partly due to the presence of a nondividing, antibiotic-tolerant subpopulation. Here we evaluated treatment of S. aureus UAMS-1 biofilms with HT61, a quinoline derivative shown to be effective against nondividing *Staphylococcus* spp. HT61 was effective at reducing biofilm viability and was associated with increased expression of cell wall stress and division proteins, confirming its potential as a treatment for S. aureus biofilm infections.

## TEXT

Antimicrobial-tolerant Staphylococcus aureus biofilms are commonly associated with chronic infections, particularly of the skin and soft tissue (1, 2). Biofilms are highly heterogeneous, containing cellular subpopulations that are nondividing and/or are metabolically inactive. As a large proportion of clinically administered antimicrobials target actively dividing cells, this adopted quiescent state renders these antimicrobials ineffective, thus allowing biofilm bacteria to survive therapeutic intervention and contribute to chronic disease (3). Ineffective treatment can also promote the evolution of resistance mechanisms within bacterial populations. In S. aureus, commonly evolved resistance mechanisms can render β-lactams such as penicillin and glycopeptides such as vancomycin ineffective (methicillin-resistant S. aureus [MRSA] and vancomycin-resistant S. aureus [VRSA], respectively) (4, 5). The combination of biofilm tolerance and evolved resistance mechanisms means that the development of novel antimicrobials targeting biofilm bacteria is highly desirable.

HT61 is a quinoline derivative that has demonstrated efficacy against both dividing and nondividing planktonic cultures of *Staphylococcus* spp. (6–8). HT61 preferentially binds to anionic staphylococcal membrane components, causing structural instability within the membrane and cell depolarization (6, 8). Given its effectiveness against nondividing cells, HT61 represents an ideal candidate for targeting the dormant subpopulations present in S. aureus biofilms.

In this study, we investigated the efficacy of HT61 against established *in vitro* S. aureus biofilms. We also utilized a quantitative label-free proteomic approach to identify changes in protein expression following treatment of planktonic and biofilm cultures with subinhibitory and inhibitory concentrations of HT61 to further elucidate the cellular processes linked to HT61’s mechanism of action. Understanding its mechanism of action further could provide insight into effective treatments for biofilm-associated chronic infections.

S. aureus UAMS-1, a methicillin-sensitive osteomyelitis isolate (9), was used in all experiments. Levels of susceptibility of planktonic and biofilm cultures of S. aureus to a range of HT61 (Helperby Therapeutics) and vancomycin (Hospira Inc.) concentrations (0.5 to 128 mg/liter) were compared. HT61 is being developed as a topical agent, and vancomycin has been used extensively as a successful topical treatment for chronic wounds and acute surgical site infections (10–12). All experiments were performed in tryptic soy broth (TSB; Oxoid), using a starting inoculum of 10^5^ cells ml^−1^, diluted from an overnight culture. All cultures were performed at 37°C with agitation (planktonic cultures, 120 rpm; biofilm cultures, 50 rpm).

Planktonic MICs were obtained using the broth microdilution method (7), and minimum bactericidal concentrations (MBCs [concentrations eliciting a 99.9% reduction in viability]) were obtained after subsequent plating and CFU enumeration on tryptic soy agar (TSA; Oxoid). Biofilm MBCs were calculated as described previously by Howlin et al. (2015) (13). Briefly, biofilms were cultured in Nunc-coated 6-well plates (Thermo Fisher, United Kingdom) for 72 h, with medium replacements every 24 h prior to antibiotic treatment. Following 72 h, spent medium was replaced with TSB containing the appropriate antibiotic dilution. Biofilms were incubated for a further 24 h. The medium was then removed, and the biofilms were rinsed twice with Hanks balanced salt solution (HBSS) to remove nonadhered cells. The biofilms were then detached and suspended in 1 ml HBSS using a cell scraper. Suspensions were serially diluted and plated onto TSA, and CFUs were enumerated following a final 24-h incubation.

**FIG 1:**
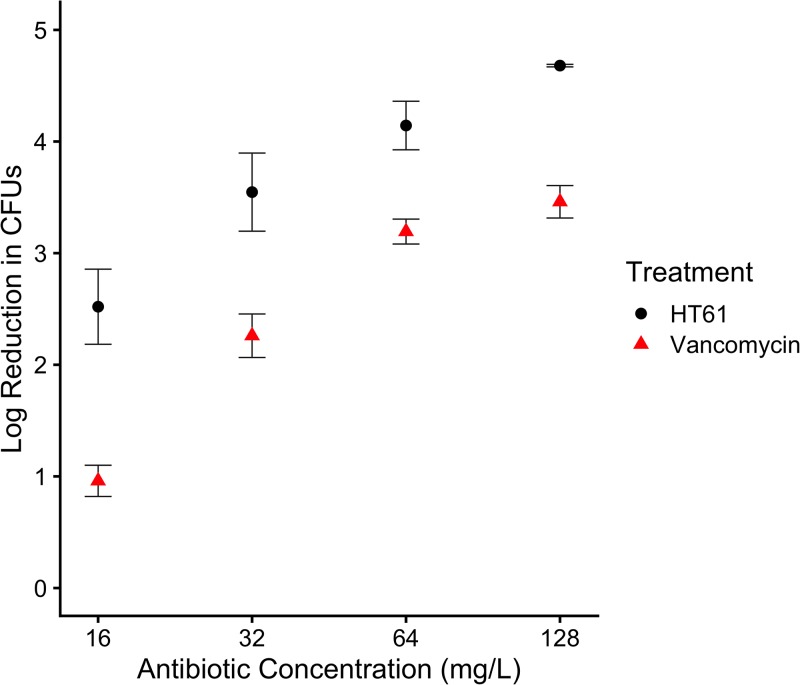
Log reduction in S. aureus UAMS-1 viable counts of an established 72-h biofilm following treatment with HT61 and vancomycin. HT61 consistently elicited a greater log reduction in CFU counts than vancomycin, demonstrating its potential as an antibiofilm agent. A higher value indicates a greater log reduction in CFU. *n* = 3. Error bars indicate standard deviations. Statistical analyses were performed using R version 3.6.0, and figure data were plotted using ggplot2 and cowplot (25–27).

The planktonic MIC and MBC values for HT61 were 16 mg/liter and 32 mg/liter, respectively, in comparison to 4 mg/liter for both the vancomycin MIC and MBC. In assays of biofilms, HT61 presented with improved killing of S. aureus UAMS-1 biofilms compared to vancomycin, demonstrated by a biofilm MBC half that of vancomycin (32 mg/liter compared to 64 mg/liter). At the maximum concentration tested (128 mg/liter), HT61 caused a further 1.3 log reduction in CFUs compared to vancomycin utilized at the same concentration (Fig. 1). The mechanism of action for vancomycin necessitates active cell wall turnover (14), so it is possible that its reduced biofilm efficacy can be attributed to the presence of a dormant cell subpopulation. As HT61 was equally effective against biofilms and planktonic cultures, this may suggest that its previously described activity against nondividing cells (6–8) confers an advantage against the biofilm phenotype.

The cellular response of planktonic and biofilm cultures following treatment with 0, 4, or 16 mg/liter HT61 was then investigated using ultraperformance liquid chromatography-mass spectrometry^Elevated Energy^ (UPLC/MS^E^). These HT61 concentrations were chosen because they were below the calculated planktonic and biofilm MBCs. Use of higher concentrations would have been highly bactericidal and would have led to the accumulation of dead cells and unwanted noise within the proteome data sets. Full details of the proteomic methods, including the method of protein isolation and the instrument settings utilized, can be found in the Text S1 in the supplemental material. Briefly, planktonic cultures were grown in TSB for 12 h at 37°C with the appropriate HT61 concentrations. Biofilms were cultured for 72 h as described above prior to replacement of the used media with TSB supplemented with HT61 at the same concentrations. Biofilms were then incubated for a further 12 h before being harvested and suspended into 1 ml HBSS. Following mechanical lysis of the cells, proteins were extracted, purified, and normalized to a final concentration of 0.25 μg/μl in 3% acetonitrile–0.1% formic acid (vol/vol).

Prepared samples were analyzed using a Waters Synapt G2Si high-definition mass spectrometer coupled to a nanoAcquity UPLC system using 4 μl of peptide extract. Processed data were searched against the Uniprot S. aureus MN8 reference database (accessed 25 January 2018) and further analyzed using a combination of UniProt database searches (www.uniprot.org; accessed between 1 May 2018 and 7 July 2018) and gene ontology analysis using GeoPANTHER (15). Each data set (see Table S1 and Table S2 in the supplemental material) was normalized to the top 200 most abundant proteins (per nanogram), and the proteins were judged suitable for quantitative analysis if the following inclusion criteria were met: presence in all 3 biological replicates, false-discovery rate (FDR) of ≤1%, and sequence coverage of ≥5%. Differential expression was defined as an expression fold change of ≥1.5 and ≤0.667 with a *P* value of ≤0.05, calculated using a one-tailed Student's *t* test.

A total of 1,448 proteins were identified across planktonic and biofilm cultures. For HT61-treated planktonic cultures, 568 (4 mg/liter) and 495 (16 mg/liter) proteins met the inclusion criteria for quantitative analysis. For HT61-treated biofilm cultures, 461 (4 mg/liter) and 498 (16 mg/liter) proteins met the inclusion criteria (Table 1). HT61 treatment resulted in the differential expression of proteins involved in a variety of functions, including cell wall biosynthesis, DNA synthesis, and metabolism (see Tables S1 and S2). Interestingly, the levels of metabolic processes were generally decreased, which may have represented an attempt by the cell to limit HT61 damage, similar to the proteomic response of methicillin-sensitive S. aureus (MSSA) to oxacillin (16).

**TABLE 1:**
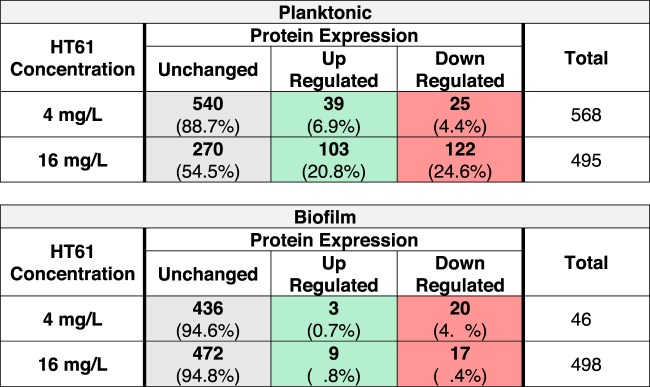
Summary of differential protein expression between untreated, sub-MIC-treated, and MIC-treated S. aureus planktonic and biofilm cultures^*a*^

a Inclusion criteria for quantitative analysis and comparison were set at 3 peptide matches, false-discovery rate (FDR) of ≤1%, and sequence coverage of ≥5%, with *P* values of ≤0.05. Data highlighted in gray, green, and red represent numbers (percentages) of samples with unchanged, upregulated, and downregulated expression, respectively. MIC, 16 mg/liter; sub-MIC, 4 mg/liter.

Treatment of planktonic cultures with a sub-MIC concentration of HT61 (4 mg/liter) revealed upregulation of MurD and MurI, two cell wall biosynthesis-associated proteins required for the incorporation of d-glutamate into cell wall peptidoglycans (17) (Table 2). Increasing the concentration of HT61 from 4 mg/liter to 16 mg/liter led to upregulation of 93% (14/15) of the proteins associated with cell wall biosynthesis, including 6 components of the mur ligase pathway (MurACDEFI; mean 2.63-fold increase) and of FemA-like protein and FemB, which are required for peptidoglycan cross-linking (mean 2.53-fold increase), and 2.19-fold upregulation of VraR, the regulator of the cell wall stress (CWS) stimulon, which is activated following the application of stress to the cell envelope (18). Proteins associated with DNA synthesis were also affected by HT61 treatment (Table 2). Subinhibitory treatment of planktonic cultures led to increased expression of DnaA and DnaX, indicating a general rise in DNA synthesis (mean 1.84-fold increase). Cell cycle-associated proteins FtsA and Obg were also upregulated (mean 2.35-fold increase), and four cell cycle-associated proteins (GpsB, GroL, Tig, and DivlVA domain protein) were downregulated (mean 0.28-fold decrease). Treatment with 16 mg/liter HT61 led to increased expression of proteins associated with DNA maintenance, including three protein with helicase activity (PcrA, GyrA, and ParE).

**TABLE 2:**
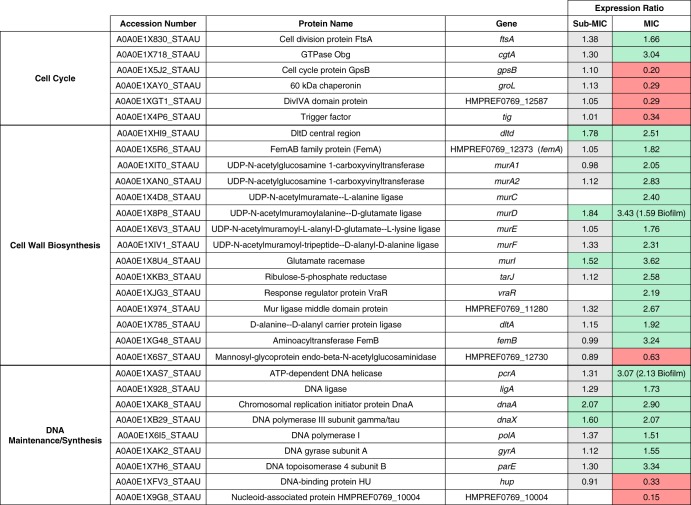
Differentially expressed proteins associated with the *dcw* and cell wall stress stimulon in S. aureus following treatment of planktonic cultures with HT61^*a*^

a Expression ratios reflect changes in expression between untreated cultures and those treated with either sub-MIC (4 mg/liter) or MIC (16 mg/liter) levels of HT61. Differential expression in biofilms is indicated in parentheses. Differential expression is defined as a fold change of ≥1.5 for upregulation (green cells) and ≤ 0.667 for downregulation (red cells). Gray cells indicate no change in expression. Empty cells represent proteins not identified.

Biofilms treated with HT61 presented with a similar, albeit more muted response (Table 1). Notably, following treatment with HT61 at 16 mg/liter, increased expression was observed for both MurD (1.59-fold) and PcrA (2.13-fold), similarly to that observed with the planktonic cultures (Table 2). It is possible that the response across both planktonic and biofilm cultures was a result of SOS response activation. The SOS response is activated upon DNA damage, and due to its quinolone-like structure, it is possible that HT61 moonlights as a DNA gyrase inhibitor or as another SOS-response inducer, leading to a cellular response much like that induced by quinolone antimicrobials, such as ciprofloxacin (19–21).

As well as being part of the CWS stimulon, a number of the differentially expressed cell wall biosynthesis components, DNA synthesis/maintenance genes, and cell cycle components comprise a segment of the division cell wall gene (*dcw*) cluster, a family of genes that are vital for maintaining cell shape and integrity (22, 23). Previous studies have shown that HT61 preferentially binds to anionic phospholipids in the S. aureus cell membrane, in a manner similar to that seen with the lipopeptide antimicrobial daptomycin (8, 24, 25). Daptomycin inserts into the cell membrane, leading to alterations in membrane curvature, potassium efflux, and membrane depolarization (24, 25), with membrane curvature shown previously to impair cell wall synthesis by affecting the MurG cell wall biosynthesis protein (26). In addition, transcriptional profiling has also shown that daptomycin upregulates components of the cell wall stimulon, suggesting a secondary mechanism of action and/or interactions with the associated components (27). Altered expression of the *dcw* cluster has also been documented in biofilms of Haemophilus influenzae following d-methionine treatment, contributing to altered cell morphology (22). It is possible that HT61 functions in a manner similar to these examples, either by directly interfering with cell wall biosynthesis machinery or by placing stress directly on the cell membrane, interfering with the cell wall machinery.

To conclude, we have demonstrated that HT61 is more effective than vancomycin at treating *in vitro* biofilms of S. aureus, although whether this translates to efficacy *in vivo* needs to be determined. Furthermore, the safety and tolerated dose of HT61 will need to be evaluated in order to determine whether it is a therapy superior to vancomycin in a clinical setting. We have also shown that HT61 influences the expression of the CWS stimulon and *dcw* cluster, in line with its predicted mechanism of action. Similarly to other quinoline-like compounds, it may also stimulate the SOS response.

## 

### Statistical analyses.

Statistical analyses were performed using R version 3.6.0, and figures were plotted using ggplot2 and cowplot (28–30).

### Data availability.

Proteomic data are available at the following URL: https://doi.org/10.5258/SOTON/D0619.

## Supplementary Material

Supplemental file 1

Supplemental file 2
