# Supplementary material for: Antimicrobial Activity of the Quinoline Derivative HT61 against Staphylococcus aureus Biofilms
Source: Antimicrob Agents Chemother. 2020 Apr 21;64(5):e02073-19. doi: 10.1128/AAC.02073-19 (PMC7179629; doi:10.1128/AAC.02073-19)
Supplement: Supplemental file 1 [file AAC.02073-19-s0001.pdf]

Response of *S. aureus* biofilms to HT61

Supplementary Methods

Planktonic and Biofilm Culture

To elucidate the cellular response towards HT61 changes in protein expression were investigated using liquid chromatography mass spectrometry$^{\text{Elevated Energy}}$, (UPLC/$MS_E$). Planktonic cultures were grown to stationary phase in TSB for 12 hours at 37 °C with either 0, 4 or 16 mg/L HT61 (sub-MIC and MIC, respectively). Biofilms were cultured for 72 hours as described earlier, prior to replacement of used media with TSB supplemented with 0, 4 or 16 mg/L HT61 for a further 12 hours. Biofilms were harvested as described previously and suspended into 1 ml HBSS.

Cell Lysis

All cell suspensions were centrifuged at 2500 *x g* for 15 minutes and rinsed twice with 0.1 M triethylammonium bicarbonate (TEAB). Suspensions were centrifuged once more and suspended in 4 M Guanidine-HCl (in 0.1 M TEAB), then mechanically lysed using a TissueLyser LT (Qiagen) with Lysing Matrix B tubes (MP Biomedicals) at 50Hz for 20 x 30 second cycles with 30 second rest on ice between cycles).

Protein Isolation and Purification

Proteins were precipitated in ice cold EtOH overnight at -20 °C and centrifuged at 12,000 *x g* for 10 minutes. Protein pellets were resuspended in 0.1 M TEAB and 0.1 % Rapigest SF Surfactant (Waters) prior to quantification using a Qubit fluorometer (Thermo-Fisher). Proteins were reduced using 2.5 mM dithiothreitol, incubated at 56 °C for 1 hour. Iodoacetamide was added (final concentration 7.5 mM) to prevent

disulphide bond reformation and incubated at room temperature in the dark for 30 minutes prior to overnight digestion at 37 °C via the addition of 0.5 μg trypsin per sample. Trifluoroacetic acid (final concentration 0.5%) was added to each sample, before centrifugation (13, 000 *x g,* 10 minutes) and dried using a SpeedVac (Thermo-Fisher) to evaporate the solvent. Samples were resuspended in 0.5 % acetic acid, purified using a C18 solid phase extraction plate (Thermo-Fisher) according to manufacturer's instructions and eluted into 80% acetonitrile and 0.5% acetic acid. Peptide extracts were lyophilised and stored at -20 °C until required.

Peptide Analysis

Peptide extracts were re-suspended in buffer A, (3 % acetonitrile, 0.1 % formic acid (v/v)) at a concentration of 0.25 μg/μL containing the internal digest standard, yeast enolase (Waters) at a final concentration of 0.25 fmol/μL. Prepared samples were analysed using a Waters Synapt G2Si high definition mass spectrometer coupled to a nanoAcquity UPLC system. 4 *μ*l of peptide extract was injected onto a C18 BEH trapping column (Waters) and washed with buffer A for 5 min at 5 *μ*l/min. Peptides were separated using a 25 cm T3 HSS C18 analytical column (Waters) with a linear gradient of 3-50 % acetonitrile + 0.1 % formic acid over 50 minutes at a flow rate of 0.3 *μ*l/min. Eluted samples were sprayed directly into the mass spectrometer operating in MS^E mode. Data were acquired from 50 to 2000 m/z with the quadrupole in RF mode using alternate low and elevated collision energy (CE) scans, resolution of 35,000. Low CE was 5 V and elevated CE ramp from 15 to 40 V. Ion mobility separation was implemented prior to fragmentation using a wave velocity of 650 m/s and wave height of 40 V. The lock mass Glu-fibrinopeptide, (M+2H)+2,

m/z = 785.8426) was infused at a concentration of 100 fmol/μl at a flow rate of 250 nl/min and acquired every 60 sec.

Raw data were processed using a custom package (Regression tester) based upon executable files from ProteinLynx Global Server 3.0 (Waters). The optimal setting for peak detection across the dataset was determined using Threshold inspector (Waters) and these thresholds were applied: low energy = 100 counts; high energy = 30 and a total energy count threshold of 750. Database searches were performed using regression tester and searched against the Uniprot *S. aureus* MN8 reference database (accessed 25/01/2018) with added sequence information for the internal standard Enolase. A maximum of two missed cleavages was allowed for tryptic digestion and the variable modifications were set to oxidation of methionine and carboxyamidomethylation of cysteine.

Each protein data set was normalised to the top 200 most abundant proteins (per ng). Inclusion criteria for quantitative analysis and comparison were as follows; the protein must be present in all 3 biological replicates with a false discovery rate (FDR) $\leq$ 1% and sequence coverage $\geq$ 5%. Differential expression was categorised by an expression rate of $\geq$ 1.5 and $\leq$ 0.667 with $p \leq 0.05$ using a one-tailed student t-test. Proteins were analysed using a combination of uniprot database searches (www.uniprot.org, accessed between 01/05/18 and 07/07/18) and gene ontology analysis using GeoPANTHER(1).

References

1. Mi H, Huang X, Muruganujan A, Tang H, Mills C, Kang D, Thomas PD. 2017. PANTHER version 11: expanded annotation data from Gene Ontology and Reactome pathways, and data analysis tool enhancements. Nucleic Acids Res 45:D183–D189.
